# Potential short-term outcome advantage of low vs. high ligation of inferior mesenteric artery for sigmoid and rectal cancer: propensity score matching analysis

**DOI:** 10.1186/s12893-023-01932-9

**Published:** 2023-02-09

**Authors:** Chia-Chen Hsu, Yu-Jen Hsu, Yih-Jong Chern, Bor-Kang Jong, Chun-Kai Liao, Pao-Shiu Hsieh, Wen-Sy Tsai, Jeng-Fu You

**Affiliations:** Division of Colon and Rectal Surgery, Department of Surgery, Chang Gung Memorial Hospital at Linkou, College of Medicine, Chang Gung University, No. 5, Fuxing Street, Guishan District, Taoyuan City, 33305 Taiwan

**Keywords:** Colorectal cancer, Inferior mesenteric artery, Propensity score matching, Low ligation, Anastomosis leakage

## Abstract

**Background:**

Whether to ligate the inferior mesenteric artery at its root during anterior resection for sigmoid colon or rectal cancer is still under debate. This study compared the surgical outcomes, postoperative recovery, and anastomotic leakage between high and low ligation of the inferior mesenteric artery through a subgroup analysis.

**Methods:**

This was a retrospective analysis of prospectively collected data. All patients who underwent colorectal resection for rectosigmoid cancer between December 2016 and December 2019 were enrolled. According to the surgical ligation level of the inferior mesenteric artery, the patients were categorized into either the high or low ligation group. The investigated population was matched using the propensity score method.

**Results:**

Overall, 894 patients with sigmoid or rectal cancer underwent elective anterior resection with high (577 patients) or low (317 patients) ligation of the inferior mesenteric artery. After the propensity score matching, 245 patients in each group were compared. High ligation of the inferior mesenteric artery was associated with higher incidence of anastomotic leakage (14.9% vs. 5.6%, P = 0.041) for mid- to low-rectum tumors and a higher incidence of complications (8.6% vs. 3.3%, P = 0.013) of grades 1–2 according to the Clavien–Dindo classification system.

**Conclusion:**

Compared with high ligation, low ligation of the inferior mesenteric artery resulted in lower likelihood of morbidity and mortality in rectal and sigmoid cancers. Moreover, low ligation was less likely to result in anastomosis leakage in mid- to low-rectal cancers.

## Introduction

Surgery remains the standard treatment for Stages I to III colorectal cancer. Radical surgery with curative intent removes the primary tumor and the drained lymph nodes.

**Fig. 1:**
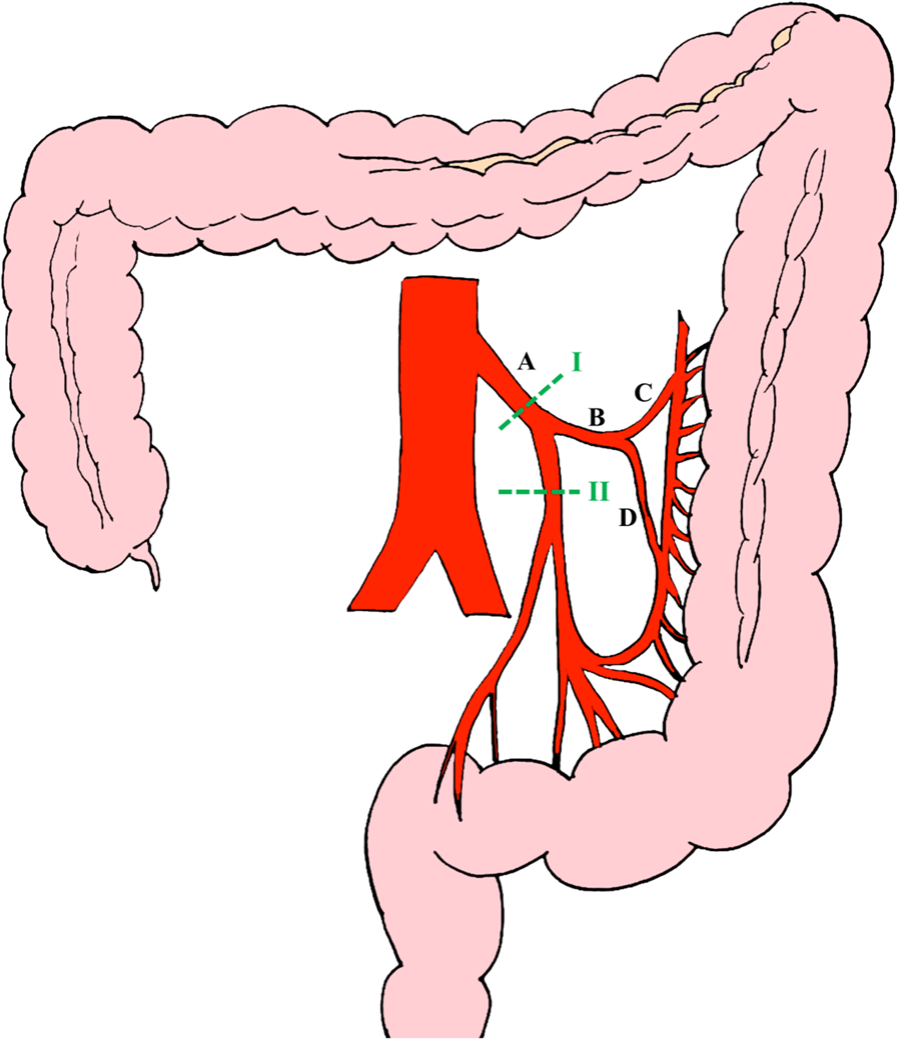
Illustration of high and low ligation of inferior mesenteric artery. **A** Inferior mesenteric artery, **B** Left colic artery, **C** Ascending branch of left colic artery, **D** Descending branch of left colic artery, I: High ligation of inferior mesenteric artery, II: Low ligation of inferior mesenteric artery

High ligation is ligation at the root of the inferior mesenteric artery (IMA), whereas low ligation is ligation distal to the bifurcation of the left colic artery (Fig. 1). Whether high ligation or low ligation should be performed for rectosigmoid cancer is still debated [1–5]. Some surgeons assume that high ligation of the IMA is crucial if extended lymph node dissection is desired because high ligation provides more accurate tumor staging and achieves better oncological outcomes [6].

High ligation of the IMA is more straightforward to perform than low ligation. Additionally, the high ligation procedure allows for maximum mobility of the remaining left colon to prevent anastomotic tension [7]. However, high ligation of the IMA has two disadvantages. First, the remaining colon segment only receives blood from the superior mesenteric artery, meaning that the blood supply in the marginal artery is diminished. Therefore, the blood supply to the anastomosis may be insufficient, especially in patients with arteriosclerosis, who may be at increased risk of anastomotic leakage [8]. Second, dissection of the IMA root may injure the superior hypogastric plexus, resulting in genitourinary dysfunction [9, 10]. By contrast, low ligation involves a delicate dissection around the IMA and preserves the left colic artery but requires more expertise in the surgeon.

Several randomized controlled studies have been designed to compare high and low ligation of the IMA to determine which technique produces better outcomes for patients with colorectal cancer. Matsuda et al. reported no statistical difference in oncological outcomes between the two approaches but did not evaluate the anastomotic leakage rate [11]. Mari et al. indicated that patients who received low ligation of the IMA had better postoperative urogenital outcomes than those who received high ligation. More patients exhibited anastomotic leakage in the high ligation group compared with the low ligation group, but this difference was nonsignificant [10]. Additionally, Feng et al. demonstrated shorter time to first flatus passage in the low ligation group than in the high ligation group [12].

The present study conducted a subgroup analysis, comparing surgical outcomes, postoperative recovery, and anastomotic leakage in high vs. low ligation of the IMA with the groups comprising propensity-score-matched patients with rectosigmoid cancer. To our knowledge, this study is the study that has enrolled the most cases of high and low ligation of the IMA through propensity score matching.

## Materials and methods

We retrieved the clinicopathological variables of patients with colorectal cancer from the Colorectal Section Tumor Registry Database of Chang Gung Memorial Hospital in Taoyuan City, Taiwan. Four nursing specialists gathered the data through patient interviews and examining clinicopathological reports, which were written using a standardized form during patient admission. The database contains several variables, including clinical characteristics, chief complaints, underlying medical illnesses, preoperative blood tests, intraoperative variables, postoperative morbidities and mortality, and tumor-related clinicopathological variables. The Institutional Review Board of Chang Gung Memorial Hospital approved this study (IRB No. 202200954B0).

### Patient selection and matched variables

This study enrolled 1325 patients who, between December 2016 and December 2019, underwent anterior resection with anastomosis for sigmoid or rectal cancer. Of these 1325 patients, 140 were excluded because they had undergone multiple surgical procedures, 109 were excluded because they had undergone noncurative resection, 33 were excluded because they received a diagnosis of Stage IV cancer, 7 were excluded because they underwent emergency surgery, and 142 were excluded because they required a diverting stoma. The 894 remaining patients, 577 who received high ligation and 317 who received low ligation of the IMA, were finally enrolled in this study.

In this study, the rectum is less than or equal to 15 cm from the anal verge and equally split into three parts: upper (10.1–15 cm), middle (5.1–10 cm), and low (0–5 cm).

### Pre-operative preparation

All patients except those with tumor obstruction underwent mechanical bowel preparation one day before surgery. Oral antibiotics are not routinely used preoperatively. All patients were delivered a prophylactic intravenous antibiotic infusion of cefazolin 1 g 30 min before the incision. If the surgery lasted more than 240 min, a booster dose of antibiotics was administered.

### Surgical technique of IMA ligation: high vs. low

A total of 18 experienced colorectal surgeons operated on all patients. Whether to perform high or low ligation of IMA was at the surgeon's discretion. The dissection plane began at the groove of the mesentery near the sacral promontory and toward the IMA root. The superior hypogastric plexus located near and behind the root of the IMA had to be recognized and separated. The IMA could often be identified and clearly defined. The IMA was ligated at its root for the high ligation group. In the low ligation group, lymph node dissection was performed around the origin of the IMA, and the dissection was continued downward distal to the root of the left colic artery. The left colic artery was identified and preserved. The IMA was then ligated and amputated distal to the source of the left colic artery.

### Propensity score matching

**Fig. 2:**
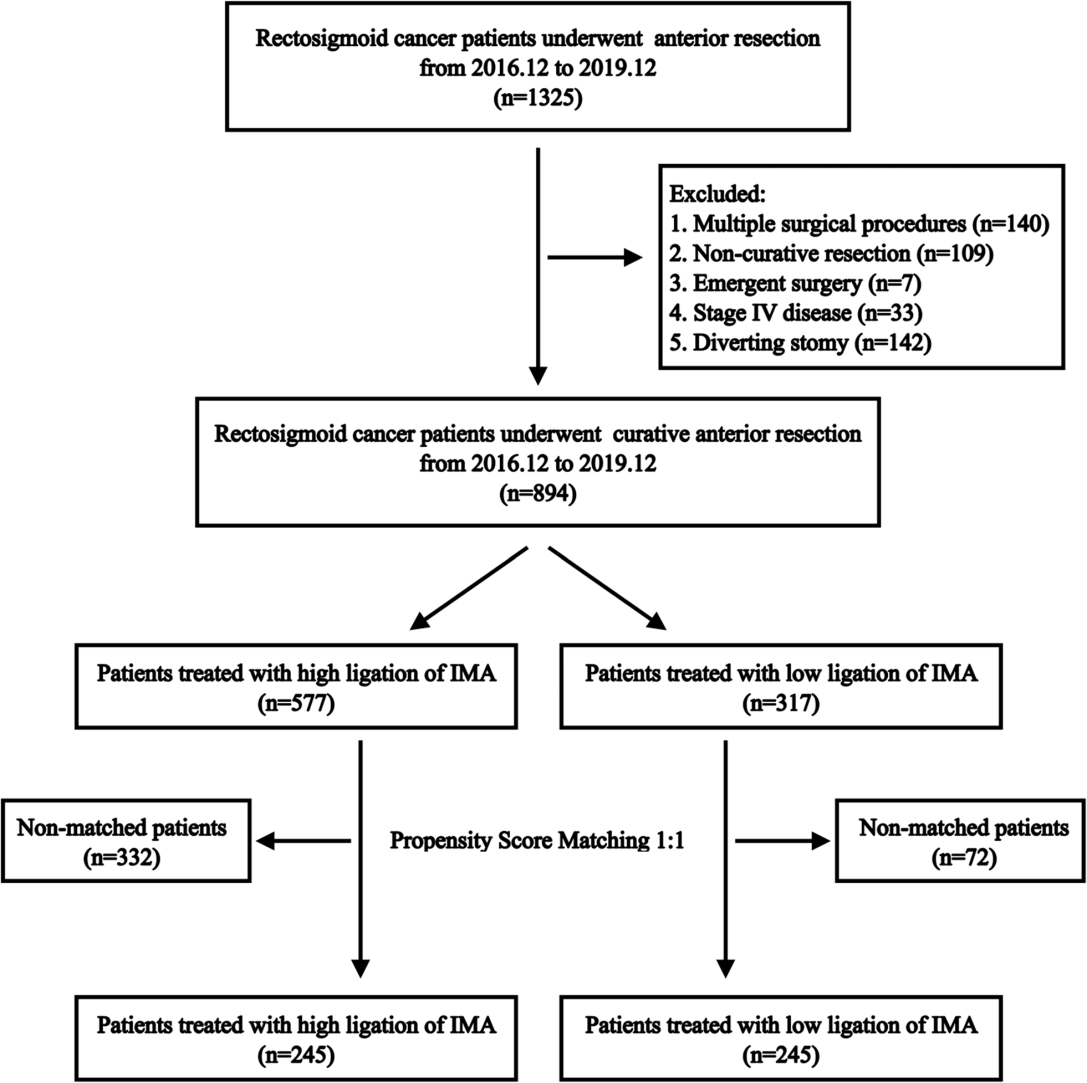
Flowchart of this retrospective study. *IMA* inferior mesenteric artery

Propensity score matching (PSM) of the two groups was conducted in the ratio of 1:1 to minimize selection bias caused by apparent differences in sample sizes and unequal covariates. The match tolerance was set at 0.02. The following variables were used for the PSM: age, gender, body mass index, American Society of Anesthesiologists (ASA) physical status score, medical illness (hypertension or diabetes mellitus), neoadjuvant therapy, preoperative laboratory data (carcinoembryonic antigen [CEA] and albumin), tumor location, tumor length, T stage, N stage, operative method, and splenic flexure mobilization. After 1:1 PSM, the number of patients in each group was 245 (Fig. 2).

### Outcomes and covariables

The measurement outcomes were short-term postoperative complications, recovery assessment, and subgroup analysis for anastomosis leakage. Postoperative complications were defined as morbidity or mortality within 30 days and were graded in accordance with the Clavien–Dindo classification system. Postoperative complications were further categorized into wound-related (wound infection or wound dehiscence), lung (atelectasis or pneumonia), cardiovascular (myocardial infarction, stroke, or embolism), urinary tract (urinary tract infection or neurogenic bladder), gastrointestinal (obstruction, ileum, or bleeding), abdomen (abscess or internal bleeding), anastomosis leakage, and other rare complications. Postoperative recovery was recorded as the length of hospital stay and the times to being able to tolerate liquid and soft diets and to first flatus and stool passages. Anastomosis leakage was defined as a defect in the integrity of the intestinal wall at the colorectal anastomotic site, with this defect enabling communication between the intra- and extraluminal compartments. A pelvic abscess near the anastomosis was also regarded as anastomosis leakage.

### Statistical analysis

All analyses were conducted using SPSS Statistics version 25.0 (IBM, Armonk, NY, USA). Clinicopathological characteristics with categorical variables are presented as frequencies and proportions and were compared using the χ^2^ test. Continuous variables were measured as mean and standard deviations and analyzed using Student’s *t* test. We performed 1:1 PSM and conducted multivariate logistic regression analysis involving multiple covariates to account for selection bias and unavoidable confounding characteristics. All statistical tests were two-tailed, and a *P* value of < 0.05 was considered significant.

## Results

### Prematching population

Overall, 894 patients with sigmoid or rectal cancer who underwent elective anterior resection with high (*n* = 577) or low ligation (*n* = 317) of the IMA between December 2016 and December 2019 were selected for analysis.

The operative parameters, recovery outcomes, postoperative morbidity and mortality, and anastomotic leakage were compared between the high ligation group and low ligation group. The baseline characteristics of the two groups are listed in Table 1. Age, gender, body mass index, ASA physical status score, medical illness (hypertension or diabetes mellitus), neoadjuvant therapy, and preoperative albumin were not significantly different between the two groups. Conversely, higher preoperative CEA, a higher proportion of sigmoid colon cancer, a more advanced T or N stage, a higher proportion of open surgery, a lower ratio of robotic surgery, and a higher proportion of splenic mobilization were discovered in the high ligation group. Tumor location and tumor stage were associated with the operative method and consequently the splenic mobilization. Moreover, the higher pre-operative CEA may have resulted from an advanced tumor stage.

**Table 1:** Baseline characteristics of high ligation and low ligation groups (before and after PSM)

	Before matching			After matching			
	High ligation (n = 577)	Low ligation (n = 317)	p value	High ligation (n = 245)	Low ligation (n = 245)	SMD	p value
Age	62.93 ± 12.13	64.91 ± 11.97	0.625	64.84 ± 11.43	64.14 ± 11.90	0.059	0.600
Gender							
Male	333 (57.7%)	182 (57.4%)	0.931	141 (57.6%)	144 (58.8%)	0.024	0.784
Female	244 (42.3%)	135 (42.6%)		104 (42.4%)	101 (41.2%)		
BMI							
< 25	336 (58.2%)	184 (58.0%)	0.957	144 (58.8%)	146 (59.6%)	0.016	0.854
≥ 25	241 (41.8%)	133 (42.0%)		101 (41.2%)	99 (40.4%)		
ASA class							
1	93 (16.1%)	60 (18.9%)	0.548	35 (14.3%)	43 (17.6%)	0.053	0.557
2	388 (67.3%)	204 (64.4%)		171 (69.8%)	161 (65.7%)		
3	96 (16.6%)	53 (16.7%)		39 (15.9%)	41 (16.7%)		
Medical illness (CVD, DM)	271 (47.0%)	156 (49.2%)	0.520	118 (48.2%)	119 (48.6%)	0.008	0.928
Neoadjuvant therapy	20 (3.5%)	17 (5.4%)	0.173	12 (4.9%)	12 (4.9%)	0	1.000
Pre-operative laboratory data							
CEA							
< 5 ng/mL	436 (75.6%)	269 (84.9%)	0.001	209 (85.3%)	203 (82.9%)	0.066	0.459
≥ 5 ng/mL	141 (24.4%)	48 (15.1%)		36 (14.7%)	42 (17.1%)		
Albumin							
< 3.5 g/dL	23 (4.0%)	7 (2.2%)	0.158	5 (2.0%)	4 (1.6%)	0.030	0.737
≥ 3.5 g/dL	554 (96%)	310 (97.8%)		240 (98.0%)	241 (98.4%)		
Tumor location							
Sigmoid	346 (60.0%)	140 (44.1%)	< 0.001	117 (47.8%)	120 (49.0%)	0.067	0.457
Upper rectum	78 (13.5%)	45 (14.2%)		41 (16.7%)	36 (14.7%)		
Middle rectum	131 (22.7%)	114 (36.0%)		69 (28.2%)	78 (31.8%)		
Lower rectum	22 (3.8%)	18 (5.7%)		18 (7.3%)	11 (4.5%)		
Tumor length	3.63 ± 4.33	3.22 ± 7.80	0.264	3.41 ± 6.36	3.13 ± 6.35	0.044	0.835
T stage							
0	6 (1.0%)	12 (3.8%)	< 0.001	4 (1.6%)	5 (2.0%)	0.073	0.415
1	69 (12.0%)	95 (30.0%)		53 (21.6%)	63 (25.7%)		
2	106 (18.4%)	59 (18.6%)		61 (24.9%)	44 (18.0%)		
3	299 (51.8%)	136 (42.9%)		114 (46.5%)	118 (48.2%)		
4	97 (16.8%)	15 (4.7%)		13 (5.3%)	15 (6.1%)		
N stage							
0	317 (54.9%)	205 (64.7%)	0.018	148 (60.4%)	149 (60.8%)	0.004	0.964
1	177 (30.7%)	77 (24.3%)		64 (26.1%)	65 (26.5%)		
2	83 (14.4%)	35 (11.0%)		33 (13.5%)	31 (12.7%)		
TNM stage							
0	5 (0.9%)	12 (3.8%)	< 0.001	4 (1.6%)	5 (2.0%)	0.013	0.885
1	134 (23.2%)	135 (42.6%)		87 (35.5%)	93 (38.0%)		
2	178 (30.8%)	58 (18.3%)		57 (23.3%)	51 (20.8%)		
3	260 (45.1%)	112 (35.3%)		97 (39.6%)	96 (39.2%)		
Operative method							
Open	91 (15.8%)	21 (6.6%)	< 0.001	25 (10.2%)	20 (8.2%)	0.04	0.653
Laparoscopy	474 (82.1%)	278 (87.7%)		208 (84.9%)	215 (87.7%)		
Robot	12 (2.1%)	18 (5.7%)		12 (4.9%)	10 (4.1%)		
Splenic mobilization	290 (50.3%)	59 (18.6%)	< 0.001	61 (24.9%)	57 (23.3%)	0.038	0.673

PSM: propensity score matching; SMD: standardized mean difference; BMI: body mass index; ASA: American Society of Anesthesiologist; CVD: cardiovascular disease; DM: diabetes mellitus; CEA: carcinoembryonic antigen; TNM: tumor node metastasis

### Postmatching characteristics

After PSM, the study population was 490 patients, with 245 patients in each group. Table 1 illustrates the baseline characteristics of the two groups after PSM. Neither the baseline or clinical variables nor the location or stage of the tumors were significantly different between the two groups.

### Postmatching results

Table 2 depicts the operative parameters in the high and low ligation groups. The operative time, intraoperative blood loss, intraoperative blood transfusion, open conversion, and proportion of secondary operations were similar between the two groups.

**Table 2:** Operative parameters in the high and low ligation groups

	High ligation (n = 245)	Low ligation (n = 245)	p value
Surgery duration (min)	224.49 ± 64.18	209.36 ± 63.58	0.829
Blood loss (ml)	48.29 ± 43.77	38.77 ± 53.15	0.595
Blood transfusion (ml)	3.27 ± 51.11	4.04 ± 44.63	0.724
Open conversion	0	0	NA
Second operation	7 (2.9%)	8 (3.3%)	0.793

Table 3 lists the postoperative recovery outcomes in the two groups. The time to first flatus and stool passages, the time to being able to tolerate liquid and soft diets, and the length of hospital stay were slightly shorter for the low ligation group than the high ligation group, but the differences were not significant.

**Table 3:** Recovery outcomes in the high and low ligation groups

	High ligation (n = 245)	Low ligation (n = 245)	p value
Time to first flatus passage (day)	2.24 ± 1.24	2.05 ± 1.09	0.154
Time to first stool passage (day)	4.42 ± 2.13	3.98 ± 2.48	0.755
Time to tolerable liquid diet (day)	4.42 ± 3.11	3.64 ± 2.69	0.585
Time to tolerable soft diet (day)	6.18 ± 3.66	5.28 ± 3.19	0.521
Length of hospital stay (day)	8.26 ± 5.48	7.07 ± 4.14	0.136

Table 4 describes the postoperative morbidity and mortality in the high and low ligation groups. The total number of mortality events was 0 vs. 2 (0.8%) in the high ligation and low ligation groups, respectively. One died of pneumonia and the other died of acute myocardial infarction. The total proportions of morbidities and mortality—including wound infection or dehiscence (1.6% vs. 0%); pneumonia or lung atelectasis (both 0.4%); MI, stroke, or embolism (0.4% vs. 0%); bladder dysfunction (2.0% vs. 1.2%); bowel obstruction or gastrointestinal bleeding (2.0% vs. 1.2%); intra-abdominal abscess or peritonitis (0% vs. 0.4%); anastomotic leakage (5.7% vs. 3.3%); and other (1.2% vs. 0.8%)—were nonsignificant differences between the groups, but the percentage of morbidities and mortality was higher in the high ligation group than the low ligation group (13.1% vs. 7.3%, *P* = 0.037). The high ligation group experienced more events that were graded 1–2 in the Clavien–Dindo classification system than did the low ligation group (8.6% vs. 3.3%, *P* = 0.013). According to the International Study Group of Rectal Cancer criteria [13], 3 out of 14 patients in the high ligation group had grade B leakage, and 11 had grade C leakage. Among the 8 patients in the low ligation group, there was 1 case of grade B leakage and 7 cases of grade C leakage.

**Table 4:** Post-operative morbidity and mortality in the high and low ligation groups

	High ligation (n = 245)	Low ligation (n = 245)	p value	Odds ratio (95% CI)
Total mortality	0 (0%)	2 (0.8%)	0.249	NA
Total morbidity and mortality	32 (13.1%)	18 (7.3%)	0.037	0.528 (0.288–0.968)
Wound (infection, dehiscence)	4 (1.6%)	0 (0%)	0.123	NA
Lung (pneumonia, atelectasis)	1 (0.4%)	1 (0.4%)	1	1.000 (0.062–16.078)
CV event (MI, CVA, embolism)	1 (0.4%)	0 (0%)	0.500	NA
Bladder dysfunction	5 (2.0%)	3 (1.2%)	0.362	0.595 (0.141–2.518)
GI event (obstruction, bleeding)	5 (2.0%)	3 (1.2%)	0.362	0.595 (0.141–2.518)
Abdomen (abscess, peritonitis)	0 (0%)	1 (0.4%)	0.500	NA
Anastomosis leakage	14 (5.7%)	8 (3.3%)	0.191	0.557 (0.229–1.353)
Others	3 (1.2%)	2 (0.8%)	0.500	0.664 (0.110–4.009)
Clavien–Dindo classification				
Grade 1–2	21 (8.6%)	8 (3.3%)	0.013	0.360 (0.156–0.829)
Grade 3–5	11 (4.5%)	10 (4.1%)	0.823	0.905 (0.377–2.172)

*CI* confidence interval, *CV* cardiovascular, *MI* myocardial infarction, *CVA* cerebrovascular accident, *GI* gastrointestinal

**Fig. 3:**
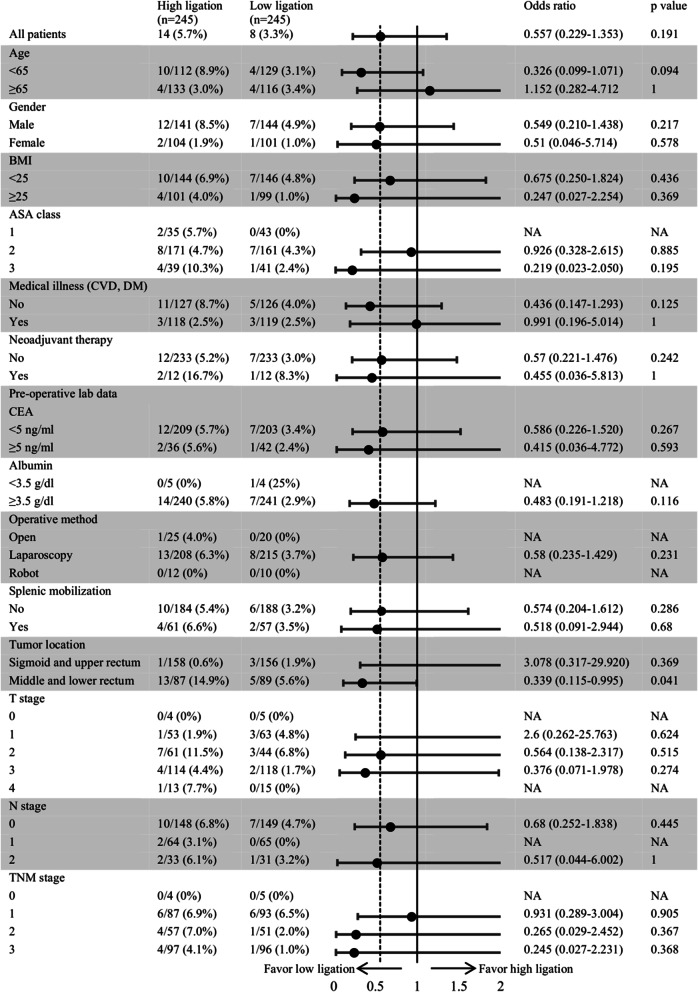
Subgroup analysis of anastomotic leakage. *PSM* propensity score matching, *SMD* standardized mean difference, *BMI* body mass index, *ASA* American Society of Anesthesiologist, *CVD* cardiovascular disease, *DM* diabetes mellitus, *CEA* carcinoembryonic antigen, *TNM* tumor node metastasis

Figure 3 illustrates the subgroup analysis of anastomotic leakage. Among age, gender, body mass index, ASA physical status score, medical illness, neoadjuvant therapy, preoperative lab data, operative method, splenic mobilization, tumor location, and TNM (tumor, nodes, and metastases) stage. For mid- to low-rectal cancers, the risk of anastomotic leakage was higher in the high ligation group than in the low ligation group (14.9% vs. 5.6%). The odds ratio (OR) for the low ligation group was 0.339 (95% confidence interval [CI]: 0.115–0.995, *P* = 0.041).

## Discussion

This study determined that overall morbidity and mortality were significantly less prevalent in the low ligation group than in the high ligation group (7.3% vs. 13.1%, *P* = 0.037). Our study collected complications in each case and classified them into eight categories that were graded using the Clavien–Dindo classification system. Although the difference in incidence for each class of morbidity was not significant, the low ligation group exhibited a trend toward lower overall morbidity, especially for Clavien–Dindo classification grades 1–2. Additionally, the risk of anastomotic leakage of mid- and low-rectal tumors was higher in the high ligation group than in the low ligation group (14.9% vs. 5.6%, *P* = 0.041).

Anastomotic leakage rates reported in various studies have differed considerably between sigmoid and rectal cancer for anterior resection of colorectal cancer. According to the literature, the anastomotic leakage rate has been reported to range from 3 to 13% [14–18]. In the present study, the overall anastomosis leakage rate was 4.5% (5.7% and 3.3% in the high and low ligation groups, respectively). However, the anastomosis leakage rate was not significant different between the high and low ligation groups (*P* = 0.191). Adequate blood supply and tension-free anastomosis are essential for successful intestinal anastomosis [19]. Some surgeons believe that high ligation, causing total obliteration of the left colic artery, affects blood flow to the end of the proximal colon stump [8, 20]. Several studies have shown that a small distance between the tumor and the anal verge is a substantial risk factor for anastomotic leakage [16–18]. Concurrent prophylactic ostomy after anterior resection certainly reduces the likelihood of symptomatic anastomotic leakage [21, 22]. We supposed that the more prophylactic ostomies are created, the more likely the rate of anastomotic leakage is underestimated. Therefore, patients who underwent concurrent prophylactic ostomy during surgery were excluded from our study. By contrast, several studies did not exclude prophylactic stoma before analysis, which may explain the inconsistent results on anastomotic leakage between high and low ligation groups. In our subgroup analysis, the incidence of anastomotic leakage in the low ligation group was lower than that in the high ligation group, for which the leakage occurred only in the mid- and low-rectum (5.6% vs. 14.9%, OR = 0.339; *P* = 0.041).

Scholars have argued whether high ligation of the IMA has the advantage of lower anastomotic tension [7, 23, 24]. A previous study using cadavers demonstrated that high ligation resulted in a 10-cm-longer colon compared with low ligation. However, low ligation combined with secondary left-colic-artery division resulted in a length of the proximal colon limb that was similar to that produced through high ligation [7]. Furthermore, the surgical technique included ligating the descending branch of the left colic artery, which increased the colon’s length and removed the tension in the anastomosis [25]. When performing low ligation, delicate vascular dissection may be required to achieve a tension-free anastomosis.

Our study discovered no differences between the two groups with regards to operative parameters, including operative time, blood loss, blood transfusion, open conversion, and second operation. Several studies have reported a longer total operative time with low ligation than with high ligation [26, 27]. Another study indicated that although the total operative time was similar for their two groups, the low ligation group had a longer operative time when IMA dissection was performed than did the high ligation group [14]. Low ligation of the IMA may have required more delicate vessel dissection than high ligation. Nonetheless, this suggests that low ligation of the IMA does not significantly prolong the total operative time because it was only a portion of the anterior resection. For most experienced surgeons, the type of IMA ligation is independent of other operating parameters, which implies that both IMA dissection techniques are safe.

Our results indicated no significant differences in postoperative recovery outcomes between the high and low ligation groups. Theoretically, bowel function impairment or genitourinary dysfunction may occur due to injury to the hypogastric plexus [10]. Other studies have noted that in high ligation of the IMA, there is a risk of injuring the superior hypogastric plexus during the IMA root dissection [9]. Zhang et al. demonstrated a shorter time to first flatus and stool passages when low ligation was performed [26]. In a study conducted by You et al., the low ligation group had a shorter hospital stay [28].

By contrast, other studies have not determined any significant differences in bowel recovery or length of hospital stay between high and low ligation groups [14, 29]. At our institute, we routinely perform lymph node dissection at the root of the IMA in patients undergoing either high ligation or low ligation to achieve satisfactory oncological outcomes. Dissection around the IMA root may lead to hypogastric plexus injury regardless of whether high or low ligation is performed. Specifically, the postoperative recovery parameters in our study were not significantly different between the two groups.

Our study has several limitations. First, this study was retrospective, and some data may have produced errors. Although the PSM method can lessen potential biases in retrospective studies, unlike the biases in randomized controlled trials, the biases caused by unobserved covariates cannot be eliminated. Second, 22.7% (72/317) of the patients in the low litigation group did not meet the established criteria and dropped out of the study after PSM, which may have threatened the validity of this analysis. Third, the actual incidence of anastomosis leakage may have been underestimated due to the exclusion of patients with a diverting stoma who received anterior resection. Nonetheless, low-rectal anastomoses were frequently accompanied by diverting stomas, which may have masked the true extent of leakage, obscuring the clinical manifestation and delaying the diagnosis. As far as we know, this is the first study using the PSM approach to perform subgroup analysis of anastomosis leakage in the high and low ligation groups. Consequently, we expect that more studies will be conducted, particularly randomized prospective studies, to verify our results and obtain additional findings on tumor location in the middle and low rectum.

This study demonstrated that low ligation of the IMA, compared with high ligation, may lead to overall lower morbidity and mortality rate in rectal and sigmoid cancers and may reduce the incidence of anastomosis leakage in mid- and low-rectal cancers while achieving comparable postoperative recovery outcomes. However, a longer follow-up period is required to determine whether long-term survival is identical. Additional case studies or prospective randomized studies are required to support our findings.

## Data Availability

The datasets used and/or analyzed during the current study are available from the corresponding author on reasonable request.

## Abbreviations

ASA: American Society of Anesthesiologists
CEA: Carcinoembryonic antigen
IMA: Inferior mesenteric artery
PSM: Propensity score matching
